# Deep Learning Predicts Subtype Heterogeneity and Outcomes in Luminal A Breast Cancer Using Routinely Stained Whole-Slide Images

**DOI:** 10.1158/2767-9764.CRC-24-0397

**Published:** 2025-01-27

**Authors:** Nikhil Cherian Kurian, Peter H. Gann, Neeraj Kumar, Stephanie M. McGregor, Ruchika Verma, Amit Sethi

**Affiliations:** 1 Department of Electrical Engineering, Indian Institute of Technology-Bombay, Mumbai, India.; 2 Australian Institute for Machine Learning, University of Adelaide, Adelaide, Australia.; 3 Department of Pathology and University of Illinois Cancer Center, University of Illinois at Chicago, Chicago, Illinois.; 4 Department of Pathology, Warren Alpert Center for Computational Pathology, Memorial Sloan Kettering Cancer Center, New York, New York.; 5 Department of Pathology and Laboratory Medicine, University of Wisconsin Carbone Cancer Center, University of Wisconsin, Madison, Wisconsin.; 6 Windreich Department of Artificial Intelligence and Human Health, Hasso Plattner Institute for Digital Health at Mount Sinai, Icahn School of Medicine at Mount Sinai, New York, New York.

## Abstract

Intratumor heterogeneity (ITH) presents challenges for precision oncology, but methods for its spatial quantification, scalable at population levels, do not exist. Based on previous work showing that the admixture of PAM50 subtype can be measured from bulk tissue using transcriptomic data, we trained a deep convolutional neural network to quantify subtype ITH in luminal A (LumA) breast cancer from routinely stained whole-slide images. We tested the hypothesis that subtype admixture detected in images was associated with tumor aggressiveness and adverse outcomes. In 680 cases from the TCGA-BRCA cohort, we determined adherence to assigned subtype by applying matrix factorization to each transcriptome. The purest cases were split into groups for initial testing, training, and parameter tuning. A total of 230 LumA-assigned cases were held out for final testing. Image patches were fed into a deep neural network pretrained on histology images. We measured the association of tumor area classified as LumA in the image with tumor characteristics and survival. Among LumA-assigned cases, admixture was associated with slightly higher estrogen receptor positivity but lower progesterone receptor (PR) positivity and estrogen receptor–related gene expression and higher HER2 positivity, tumor size, grade, and tumor–node–metastasis stage. Image admixture was associated with more *TP53* and fewer *PIK3CA* mutations. Progression-free survival was significantly shorter in more admixed cases. Our findings demonstrate that deep learning, trained to recognize genomic correlates in tissue morphology, can quantify and map subtype admixture in LumA breast cancer that has clinical significance. The low cost and scalability of this method hold potential as a research tool for investigating ITH and perhaps improving the efficacy of precision oncology.

**Significance:**

A deep learning model, trained using transcriptomic data, inexpensively quantifies and fine-maps ITH due to subtype admixture in routine images of LumA breast cancer, the most favorable subtype. This new approach could facilitate exploration of the mechanisms behind such heterogeneity and its impact on selection of therapy for individual patients.

## Introduction

Breast cancer is a complex disease with molecular and morphologic characteristics that vary not only across patients but also within the same tumor mass (1–3). To better guide prognosis and treatment, breast cancers are often classified into intrinsic subtypes using PAM50 profiling—a 50-gene signature based on bulk tissue sampling—or a surrogate IHC panel, which categorizes each case into one of four major subtypes: luminal A (LumA), luminal B (LumB), HER2, and basal (4). However, individual tumors often exhibit signs of admixture with a different subtype than the one assigned using PAM50 profiling, which suggests that PAM50 profiling may not fully capture the heterogeneity of the tumor (5, 6). Treating a cancer with the assumption of homogeneity can ignore the heterogeneous nature of individual tumors, which could lead to treatment failure (7).

The PAM50 system for classifying intrinsic subtypes has proved to be robust, in terms of its ability to subdivide cases according to prognosis and response to various therapeutic strategies (8). However, the PAM50 algorithm assigns a subtype to a case based on its location in the multidimensional space to the nearest centroid belonging to a given subtype in a reference population. Thus, subtype assignment is the same whether an individual case is very close to its assigned centroid and far from any alternate centroids versus a case that is distant from its assigned centroid and somewhat proximate to an alternative. In previous work, we showed that these relative distances for PAM50 genes can be transformed into a metric that predicts tumor aggressiveness and survival (9). Subsequently, we showed that subtype admixture in LumA can be quantified even more accurately by applying matrix factorization to the entire transcriptome to measure the degree of adherence of each tumor to its assigned class (10). Although both studies revealed strong associations of quantified heterogeneity in LumA breast cancer with tumor characteristics and outcomes, these methods rely on genomic analysis of the bulk tissue, which is moderately expensive and devoid of spatial resolution. Methods with varying degrees of spatial information, such as multiregion or single-cell sequencing and spatial transcriptomics, currently have limitations because of sampling concerns, scalability, and ease of adoption (11, 12).

The existence of distinct intrinsic subtypes implies that each subtype represents a favorable genomic arrangement for promoting tumor growth. This further implies that subclones arising within a single tumor, conceivably in response to stochastic mutation or a difference in the local microenvironment, could adopt an alternative subtype program (13). The plausibility of subtype heterogeneity within a single tumor is indirectly supported by multiple lines of evidence, including spatially discordant expression of established markers such as estrogen receptor (ER), PR, and HER2, discordance between PAM50- and IHC-based classifications, genomic differences between a primary tumor and metastases, and decreased responsiveness to anti-HER2 therapy in HER2-positive tumors that also express ER (14–16). Therefore, more nuanced diagnostic approaches that consider the potential for tumor heterogeneity and the coexistence of alternate subtypes are needed to improve the accuracy of prognosis and treatment guidance for patients with breast cancer.

Here, we propose a method of quantifying subtype heterogeneity in LumA breast cancer directly from whole-slide images (WSI) of slides routinely stained with hematoxylin and eosin (H&E). This inexpensive method can reveal the heterogeneity of a single tumor at a fine spatial resolution, which can aid in treatment planning as well as directing tissue sampling for sequencing. Our approach utilizes the proportions of subtypes derived from the factorization of the transcriptome to identify relatively homogeneous WSIs for training a deep convolutional neural network (DNN) and selects a model in which predictions maximally correlate with subtype admixture proportions on held-out tumor images. We tested the hypotheses that (i) transcriptomes strongly linked to intrinsic subtype have corresponding phenotypes that can be spatially detected in WSIs by an appropriately trained DNN and (ii) heterogeneous LumA images, as revealed by a DNN, have worse tumor characteristics and outcomes than homogeneous ones.

## Materials and Methods

### Study population and data sources

**Figure 1:**
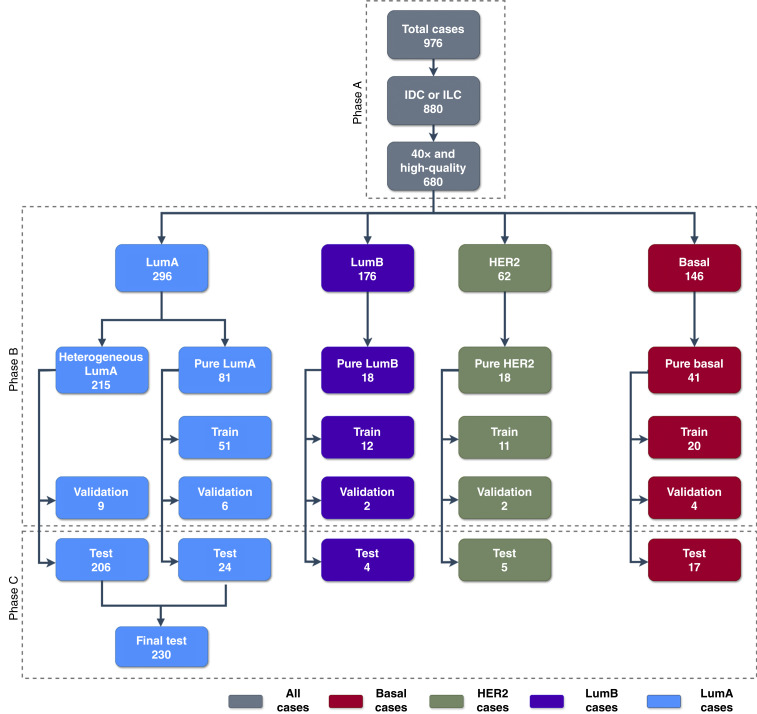
Diagram depicting the allocation of breast cancer WSIs for analysis. Phase A: filtering of TCGA images. Phase B: 680 cases were divided by assigned subtype, and then subsets with transcriptomically pure subtype adherence were randomly split into training, validation, and test subgroups. Phase C: Pure cases for each subtype were held out for initial testing. The final test set used all LumA cases that were held out, representing the full range of LumA transcriptomic purity. IDC, invasive ductal carcinoma; ILC, invasive lobular carcinoma.

We downloaded WSIs, genomic data, and clinical data from Genomic Data Commons for 976 breast cancers in The Cancer Genome Atlas (TCGA) cohort (17). As shown in Fig. 1 (phase A), we filtered these to include 880 cases labeled as either invasive ductal or invasive lobular. Further restriction to images obtained at 40× that were deemed to be of adequate quality left 680 cases for potential inclusion in the study. Based on the assigned PAM50 class (excluding the normal subtype), this study comprised 296 LumA, 176 LumB, 62 HER2, and 146 basal cancers. These cases were ranked for subtype purity using the method described below, which determines purity by transcriptomic adherence to the assigned subtype. All 296 LumA cases, plus the most highly ranked cases among the other 3 assigned subtypes, were used in DNN model development, as shown in Fig. 1 (phase B). Tissues used to produce WSIs were collected prospectively from multiple clinical centers using TCGA protocols and were shipped to specialized centers for slide preparation and scanning.

### Quantifying subtype purity

We applied semisupervised nonnegative matrix factorization (ssNMF) to expression data for 11,379 genes to compute the subtype admixture proportions of the four major subtypes—denoted as pLumA, pLumB, pHER2, and pBasal—for each case. This method models the transcriptomic data as an admixture of the underlying metagenes (18). We normalized the data so that the four proportions summed to one for each case; see reference 10 for additional details.

### Data splits

We hypothesized that the visual cues related to intrinsic subtype can be better learned by a DNN if the training data came from relatively pure samples. Therefore, we selected training samples that exhibited the largest contribution to their transcriptomic signature from their assigned subtype according to the ssNMF model. As shown in Fig. 1 (phase B), we selected the 81 highest ranked (purest) LumA cases and 77 of the purest cases that were PAM50-assigned to other subtypes to be used for training, validation (hyperparameter tuning), and initial testing of the model. A total of 94, 23, and 50 pure cases were used for training, validation, and initial testing, respectively. The 24 pure LumA cases held out for initial testing plus 206 more heterogeneous LumA cases comprised the held-out set for final testing, as shown in Fig. 1 (phase C). Cases from 18, 12, 15, and 23 distinct clinical centers were represented in the training, validation, initial (pure case), and final test sets, respectively [Supplementary Table S1 (parts A–D)]. The final test set included 17 cases from eight centers not represented in training; we analyzed this subset separately from the other test cases for external validation based on alternate tissue sources.

### DNN design and workflow

As digitized biopsy slides are high-resolution gigapixel images that cannot be directly input into a DNN, we extracted nonoverlapping patches (subimages or tiles) of size 512 × 512 pixels at 40× resolution (approximately 0.25 microns per pixel) to strike a balance between having an adequate spatial context and being able to load multiple patches simultaneously into the graphics processing unit (GPU) of the computer. Patches were extracted from tumor regions digitally annotated by a blinded breast pathologist (S.M. McGregor), focusing on the comprehensive sampling of diverse morphologies in each slide and careful avoidance of areas that demonstrated a technical artifact. The annotations helped ensure that the training data were of high quality as they avoided nontumor regions, including regions containing mostly stroma, whereas the selection of genomically pure slides for training helped avoid mixed tumor subtypes. These design features enabled strongly supervised DNN training as all the patches extracted from a WSI were given the same class label as that of the WSI itself—LumA or non-LumA. Approximately 133,000 patches were extracted from all training WSIs.

**Figure 2:**
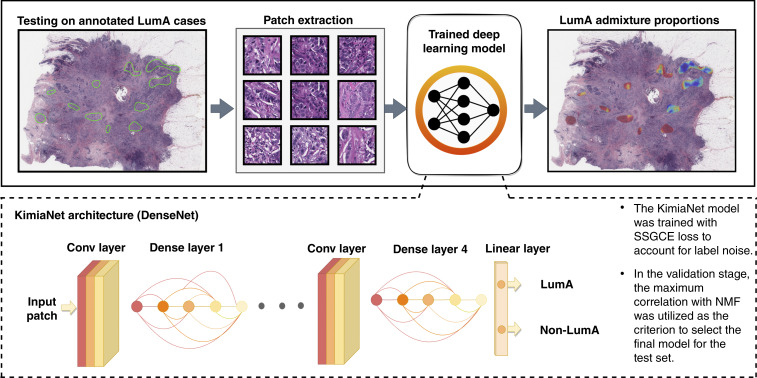
Schematic overview of the method for quantifying subtype heterogeneity in held-out whole-slide H&E images from TCGA-BRCA cohort. Conv-layer, convolutional layer; SSGCE, sample-specific generalized cross-entropy.

Patches collected after the data preparation step were used to fine-tune KimiaNet, a DNN based on the DenseNet-121 architecture with four dense blocks and more than seven million trainable parameters (19). KimiaNet, which is already pretrained on several multiorgan histopathology datasets, offers the advantage of starting with a DNN in which lower layers capture histology-specific features, unlike those pretrained only on generic image datasets, such as ImageNet. A schematic of the model architecture and workflow for final testing is shown in Fig. 2.

### Model training, validation, and testing

For training, we used the Adam optimizer at a learning rate of 10^−4^. To make the classifier robust to stain variations and prevent overfitting, we used various augmentation methods, including color jitter, rotations, flips, and random elastic deformations, along with a weight decay of 0.2. All experiments were performed on patches of 512 × 512 pixels for a batch size 80, trained on two NVIDIA v100 GPUs.

Although we increased the label quality of patches used for training by selecting transcriptomically pure WSIs and annotating tumor regions, we assumed that some patches were mislabeled because of spatial heterogeneity and the diversity of shapes and sizes of tumor nests, making the labeling inherently noisy. Cross-entropy, a popular objective function to be minimized for training neural networks, explicitly encourages overfitting on noisy labels. Therefore, we used sample-specific generalized cross-entropy, which has been shown to be more robust to label noise (20). Sample-specific generalized cross entropy uses the early training iterations to identify noisy labels and reduces emphasis on them during later iterations.

The usual method for selecting hyperparameters of a DNN is to check the classification performance of the trained model on a held-out validation set. In our case, that would be checking the accuracy of LumA versus non-LumA classification in the validation dataset. However, to obtain better calibration, we picked hyperparameters that maximized the Pearson correlation between the LumA patch proportion estimated by the DNN (denoted as iLumA%) and LumA genomic admixture proportion estimated by the ssNMF method (pLumA). The correlation approach was enhanced by expanding the validation data to include samples that were more heterogeneous than the training data.

In initial testing, we determined how well the model could discriminate between cases that were either pure LumA or pure for one of the other three subtypes. For final testing, we ran the model on 230 LumA cases that had not been used in training or validation to determine the tumor area classified as LumA. We then repeated this analysis on a subset of cases obtained from hospitals excluded from training.

### Data analysis

We calculated overall accuracy at the patient level, using majority voting based on patch classifications, to evaluate the ability to discriminate pure LumA cases from pure non-LumA in the initial testing set. We used scatterplots to graphically compare the joint distribution of iLumA% and pLumA for these relatively pure cases. For the final test set, we computed Pearson and Spearman correlation coefficients between iLumA% and pLumA. We evaluated the association of iLumA% with selected clinicomolecular characteristics by dividing cases into quartiles and using either χ² or Student *t* tests to compare the highest quartile (purest) with the lowest (admixed). We also fit linear regression models across quartiles, with quartiles coded ordinally, to test for linear trends.

Clinical variables included mean age at diagnosis, percentage with nodal involvement, tumor size >20 mm, stage >I, and ER, PR, or HER2 positivity (by IHC and/or FISH). As tumor grade was not available, a single pathologist coauthor (S.M. McGregor), blinded to model results and clinical outcomes, graded each case using Nottingham score criteria. We used published formulas and normalized gene expression data to recompute the PAM50 11-gene proliferation score, PAM50 risk-of-recurrence score, Oncotype DX score, and high-risk designation by MammaPrint (21). We used the four genes in the Oncotype DX ER group (*ESR1*, *PR*, *BCL2*, and *SCUBE2*) to compute an ER-related gene expression score. We obtained mutational load data from Genomic Data Commons based on whole-exome sequencing of more than 15,000 genes and computed the MATH score—a measure of mutant allele heterogeneity developed in head and neck cancer (22). Finally, we evaluated somatic mutations in *TP53*, *PIK3CA*, and *CBFB*, which are typically negatively or positively associated with LumA status.

To compare progression-free survival (PFS) and overall survival (OS) between groups based on iLumA%, we used Kaplan–Meier curves and log-rank statistics. In addition, we fit Cox proportional hazards models to estimate HRs and 95% confidence intervals, both with and without covariates. To visualize intratumor heterogeneity (ITH), we generated heatmaps by overlaying the classification probability of a patch and stitching the individual patches back to the original WSI. Patch edges were smoothed using an isotropic 2D Gaussian filter with an SD of 2.5. All *P* values were two-sided.

### Data availability

Data and code used in this study are accessible in GitHub (https://github.com/nikhilkurian/iLumAScore).

## Results

**Figure 3:**
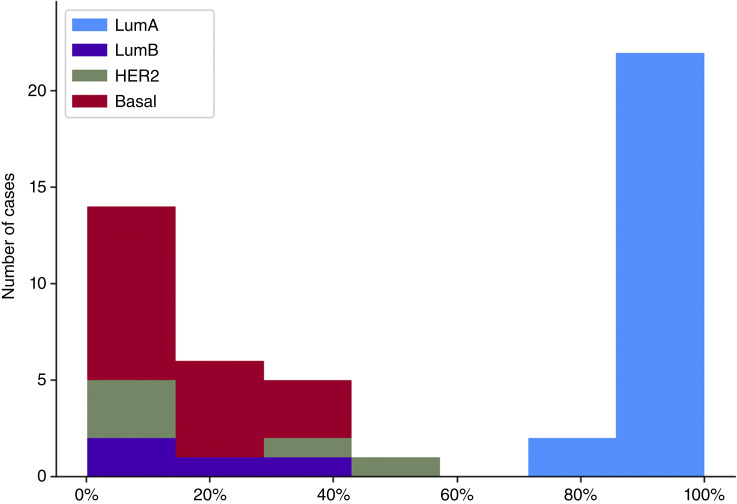
Frequency distribution for the percentage of image patches assigned to LumA by the DNN model among the held-out transcriptionally pure cases grouped by PAM50-assigned subtype. The numbers of patches analyzed for LumA, LumB, HER2, and basal were 10,942, 4,914, 5,749, and 11,172, respectively.

**Figure 4:**
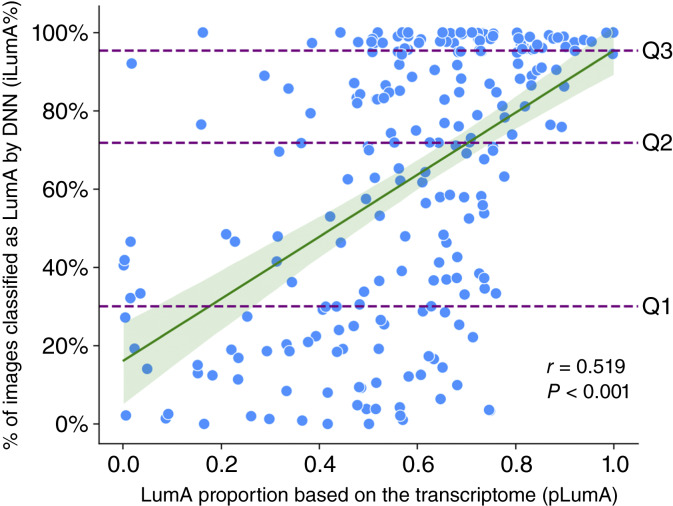
Scatterplot of the LumA proportion by transcriptomic analysis vs. percentage of tumor image patches classified as LumA by the DNN model. *N* = 230 cases assigned to LumA by PAM50. A best-fitting regression line is shown, with 95% confidence band. Horizontal lines depict quartile thresholds for the number of cases.

In the initial test, the final model discriminated with 100% accuracy between held-out LumA and non-LumA cases that showed strong adherence to their assigned subtype based on transcriptomic profiling. Figure 3 shows the frequency distribution for iLumA%, the proportion of patches classified as LumA, stratified by assigned PAM50 subtype. Figure 4 shows the correlation in the LumA-assigned test set between subtype admixtures based on the transcriptome alone versus the admixture detected in the image. The Spearman correlation among the 230 held-out cases assigned to LumA was 0.52 (*P* < 0.001), but considerable deviation from the regression line is evident. A similar plot that includes all held-out cases, including those with pure transcriptomes reflecting their PAM50 assignment to LumB, HER2, and basal, had a correlation of 0.60 (*P* < 0.001) and is shown in Supplementary Fig. S1.

The associations of clinical and molecular characteristics with quartiles of LumA patch proportions in the LumA-assigned test set are shown in Table 1. (Similar results for the association of these characteristics with transcriptome-based admixture are shown in Supplementary Table S2.) These results reveal strong signs of heterogeneity within LumA-classified tumors. Relative to the most admixed cases in Q1, the purest cases in Q4 were on average 3.74 years younger at diagnosis. The percentage of ER-positive cases was generally high but trended slightly downward as the proportion of image patches classified as LumA increased. In contrast, the PR-positive percentage trended upward as iLumA% increased, as did the ER gene group score from RNA sequencing. More than a third of the most admixed cases were HER2 positive by IHC or FISH, with prevalence less than half as high in the purer quartiles. As a result, there was a strong positive trend between image purity and the prevalence of surrogate criteria for LumA status (ER or PR positive, HER2 negative; ER and PR positive, HER2 negative).

**Table 1:** Clinical and molecular features of PAM50 LumA breast cancers in the test set according to the quartile of the tumor area classified as LumA by the deep learning model (total *n* = 230)

Feature	Q1	Q2	Q3	Q4	*P* (Q1 vs Q4)	*P* trend
Age (mean, years)	58.27	57.54	57.78	54.53	0.021	0.163
ER^+^ (%)^a^	100.00	98.18	94.33	93.02	0.021	0.017
PR^+^ (%)	82.22	89.28	91.07	95.35	0.007	0.027
HER2^+^ (%)^b^	36.66	15.38	12.50	14.81	<0.001	0.217
ER or PR^+^/HER2^−^ (%)	66.67	77.50	81.39	86.20	0.002	0.029
ER and PR^+^/HER2^−^ (%)	63.27	76.92	76.08	79.55	0.018	0.148
ER gene group score (mean)	8.94	9.20	9.16	9.45	0.001	0.079
Node positive (%)	54.72	60.00	50.00	50.88	0.707	0.390
Tumor size >20 mm (%)	57.78	50.70	44.61	39.22	0.011	0.002
Grade 3 (%)^c^	33.33	18.75	16.32	8.33	<0.001	0.042
TNM stage >1 (%)	78.33	69.64	72.74	60.38	0.009	0.127
Proliferation score (mean)^d^	8.72	8.18	8.28	8.15	<0.001	0.213
Recurrence score (mean)	36.23	29.66	31.29	29.10	0.042	0.213
Oncotype DX score (mean)	40.95	30.38	29.84	26.49	<0.001	0.094
MammaPrint high (%)	37.78	12.28	5.26	1.96	<0.001	0.042
Mutational load (median)	21.0	24.0	21.5	19.0	0.139	0.466
MATH score (mean)^e^	0.350	0.357	0.363	0.371	0.545	0.001
*TP53* mutation (%)	16.98	12.50	10.71	6.25	0.026	0.012
*PIK3CA* mutation (%)	28.30	32.14	37.50	50.00	0.002	0.038
*CBFB* mutation (%)	5.66	3.57	5.35	9.25	0.150	0.319

Abbreviation: TNM, tumor–node–metastasis.

a ER and PR determined by IHC.

b HER2 determined by IHC or FISH.

c Nottingham grade determined by blind rating from a single pathologist.

d Proliferation and recurrence scores recomputed using the PAM50 method; Oncotype DX score and MammaPrint high-risk were recomputed.

e MATH score, see reference (22).

We observed significant inverse associations between purity detected in the image and tumor size, high tumor grade, and stage greater than 1. For example, the most admixed cases were more than 18% more likely to be larger than 20 mm, and the prevalence of grade 3 in Q1 was four times higher than in Q4. A small decrease in node positivity in Q3 and Q4 was compatible with chance. Observations on molecular variables indicated significant inverse associations between image purity and the PAM50 proliferation and recurrence scores. Remarkably, nearly 38% of the Q1 cases had high-risk scores based on MammaPrint genes versus less than 2% of the cases in Q4. Mutational load showed no association with image purity. The MATH score was also uncorrelated with image purity, despite a statistically significant but negligible positive trend across patch means. In contrast, the associations of image purity with *TP53*, *PIK3CA*, and *CBFB* mutations were perfectly aligned with prediction based on established relationships between these mutations and LumA status by PAM50. Mutations in *TP53* were nearly three times more prevalent in Q1 versus Q4, whereas *PIK3CA* and *CBFB* mutations, which are enriched in LumA, were approximately half as prevalent.

Supplementary Figure S2 shows scatterplots comparing results for test cases from hospitals excluded from training versus all other test cases. Importantly, Supplementary Fig. S2A shows similar slope and correlation between iLumA% and pNMF, indicating the ability of the model to convert transcriptomic data to images from sources external to training. Similar relationships were seen for Oncotype DX and MammaPrint scores (Supplementary Fig. S2B and S2C). The model detected frequent admixture with HER2; in the least pure quartile of iLumA% among test cases, the median transcriptome admixture with HER2 was 14%. Supplementary Figure S2D shows that expression of *GRB7*, which is often co-amplified with HER2 and is an established mediator of HER2 activity, had similar associations in both subsets (23, 24).

**Figure 5:**
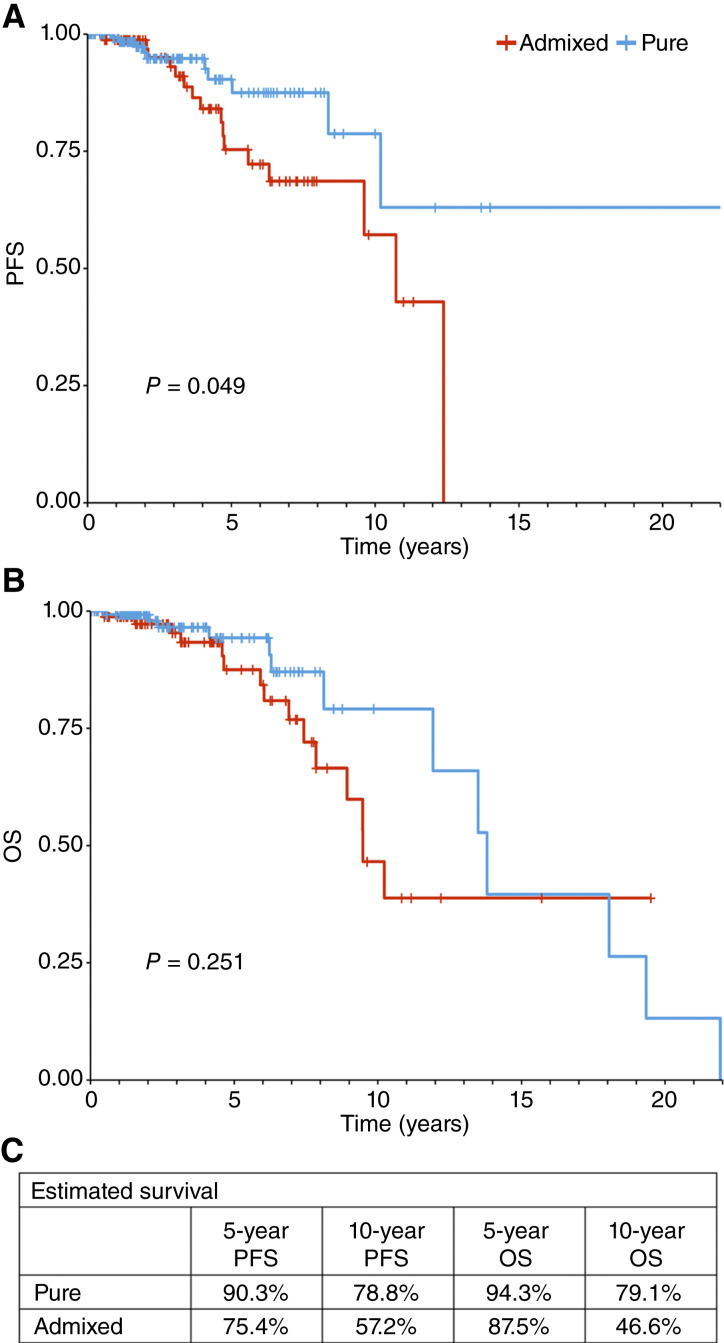
Survival plots illustrating PFS (**A**) and OS (**B**) of the pure vs. admixed cases in the test cohort. Pure is defined as having a majority of image patches classified as LumA. Estimated survival times are shown in **C**.

**Figure 6:**
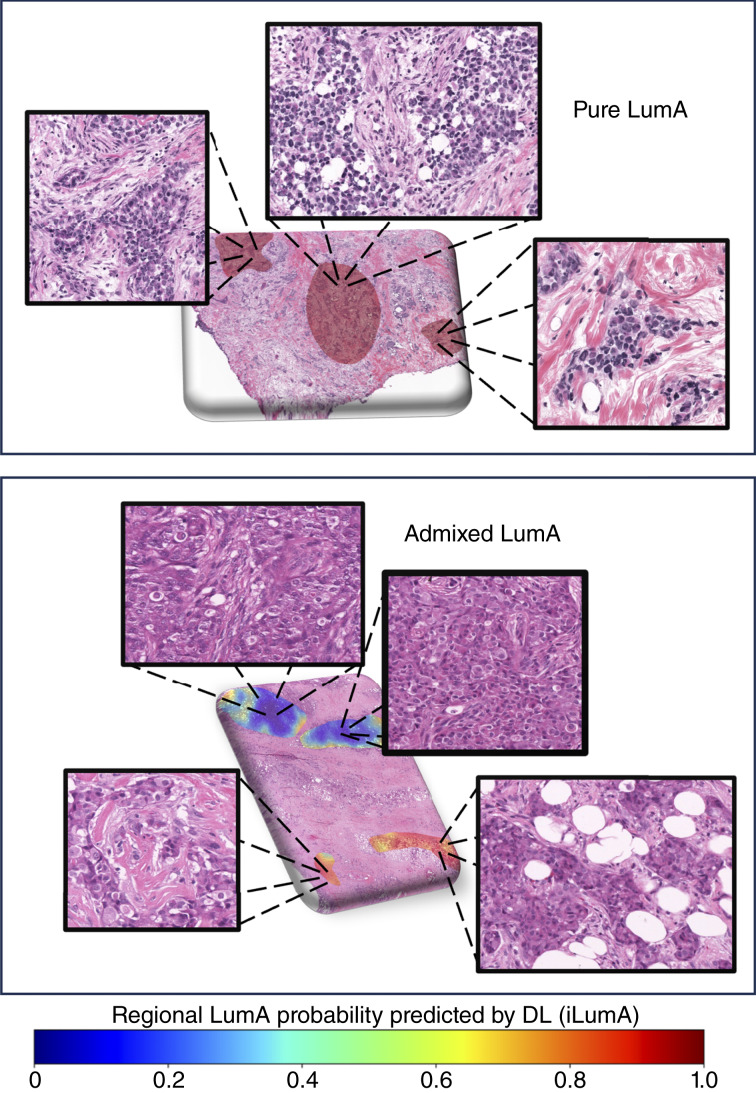
Heatmaps illustrating spatial heterogeneity in LumA subtype purity determined by the DNN model. A case with pure LumA shown at the top and admixed LumA at the bottom, with insets showing morphologic details. DL, deep learning.

PFS curves comparing admixed with pure cases in the test cohort, as determined by majority voting at the patch level, are shown in Fig. 5A. Decreased PFS is seen in the admixed group, especially after 3 years of follow-up. Similar results are shown in Fig. 5B for OS. The number of endpoints limits precision, but the same pattern is evident, with equivalent survival for the first 3 years followed by increasing separation. The estimated 10-year PFS (Fig. 5C) was 78.8% versus 57.2% for pure and admixed cases, whereas the estimated 10-year OS was 79.1% and 46.6%, respectively. HR estimates shown in Table 2 indicate that the risk of progression is approximately doubled overall in the admixed cases and that this risk ratio is more than tripled after 3 years. The subtype heterogeneity at play can be viewed spatially in heatmaps, as shown in Fig. 6, in which histomorphologic differences are evident between tumors with highly contrasting proportions of patches classified as LumA.

**Table 2:** PFS for the test cohort of PAM50-assigned LumA breast cancer, comparing pure vs. admixed cases based on the proportion of the tumor image classified as LumA by the deep learning model

	HR	95% CI	Log-rank *P*
Entire follow-up			
Pure^a^	1.00	Ref.	—
Admixed	2.18	0.98–4.82	0.049
0–3-year follow-up			
Pure	1.00	Ref.	—
Admixed	1.76	0.47–6.68	0.402
>3-year follow-up			
Pure	1.00	Ref.	—
Admixed	3.11	1.09–8.89	0.031

Abbreviations: CI, confidence interval; Ref., reference.

a Admixture defined as a majority of the tumor area classified as non-LumA.

## Discussion

We have developed a deep learning model, trained using only bulk transcriptomic data, that can recognize and quantify intrinsic subtype admixture in PAM50-determined LumA breast cancer, based on routinely available H&E-stained WSIs. To the best of our knowledge, this report is the first to reveal such ITH in a spatial context among these relatively favorable risk cases while demonstrating its links to both adverse tumor characteristics and patient outcomes. The determination of the LumA patch proportion in a tumor image was successful in stratifying patients based on the prevalence of characteristics canonically associated with either LumA or more aggressive non-LumA cancer. A critical component in the workflow was the application of ssNMF decomposition to RNA sequencing data to measure the extent to which each case adhered to its assigned PAM50 subtype and then the use of this information to label cases for model training. The correlation observed in the test cases between transcriptome adherence and a LumA pattern and the proportion of tumor classified by the model as LumA indicates that model training succeeded in translating bulk sample gene expression data to the image.

The observation that patients with relatively pure tumor images for LumA were younger than the most admixed group is consistent with our previous finding based on transcriptomic subtype purity in a combined TCGA–METABRIC population. In our previous study, we found that patients whose dominant alternate subtype was basal were in fact younger than those with pure LumA; however, basal admixture was relatively uncommon compared with patients with either dominant LumB or HER2 admixture who were older than those with pure LumA (10). Conceivably, tumors evolving in older women have more time to develop alternate subtypes. In terms of ER expression by IHC, the results show a small but significant decrease in the percentage of ER-positive cases as image purity increases, whereas transcriptome purity showed no association. Meanwhile, PR expression by IHC had clearly positive associations with both image and transcriptome purity. As PR is responsive to ER, this suggests that in pure cases, downstream ER activity is actually increased along with purity, a point further supported by our finding that the four-gene score for ER activity was significantly increased in cases with high image purity, despite the slight decrease in ER protein detection by IHC.

HER2 positivity by IHC or FISH was surprisingly common in tumors with the largest amount of area classified as non-LumA. This association was also observed to a lesser extent when admixture was determined by the transcriptome alone. It would be interesting to know if HER2 admixture, detected by our model based on histomorphology, improves the ability to identify tumors with significant ER activity that respond poorly to anti-HER2 therapy (25, 26). The null association with mutational load suggests that heterogeneity in LumA is not due to inherent genomic instability as the load score includes nonfunctional as well as functional sequence variants. Although we hypothesized that image purity and genomic heterogeneity would be inversely related, the lack of association between image purity and mutant allele heterogeneity could be due to the MATH score being DNA-based, whereas our model was trained on RNA expression. Moreover, increased genomic diversity has been reported in LumA compared with other subtypes, which could offset the heterogeneity produced by aggressive non-LumA subclones (27). The striking association of *PIK3CA* mutation with high LumA purity points to the potential importance of evaluating primary tumors for the specific alterations that have been associated with endocrine therapy resistance and responsiveness to *PIK3CA* inhibitors in a subsequent metastatic setting (28, 29).

Strong associations are evident between the degree of subtype admixture based on the image and features of aggressiveness such as tumor size, grade, and genomic risk profile. We did not see a definite relationship with nodal status at diagnosis, suggesting that the DNN model could be less effective at picking up signals related to early metastatic potential. However, the fourfold increase in the prevalence of grade 3 cancer in the most admixed tumors versus the purest and the large associations with high scores on two independent gene expression profiles validated to predict aggressiveness strongly indicate that patient outcomes are likely to be worse. The PFS and OS we observed were indeed worse in more admixed cases, notwithstanding constraints on precision because of the modest size of the test cohort. We note that survival was essentially indistinguishable in the first 3 to 5 years of follow-up, followed by significant separation. This is consistent with longstanding observations that hormone receptor–positive breast cancers are prone to late recurrence and suggest that subtype admixture can identify subgroups that are particularly susceptible (27). Hypothetically, these admixed tumors become more aggressive over time as subclones with less resemblance to prototypical LumA begin to emerge.

The remarkable power of DNNs for detecting molecular and histologic phenotypes in tumor images has been demonstrated for several cancer types, including breast cancer (30–32). Although previous DNN models have been developed specifically to recognize ITH in breast cancer histomorphology, few previous studies have used this approach to explore subtype heterogeneity itself (33). Jaber and colleagues (34) trained a DNN model on TCGA data to recapitulate PAM50 classification in H&E-stained WSIs and in the process discovered that tumors with ambiguous subtype assignment (defined as tumors in which large proportions of patches were classified as LumA and basal) had survival that was intermediate between those classified strongly as either LumA or basal. Several key distinctions between our present work and this seminal report should be noted. In that earlier work, cases were selected for training based on a PAM50 metric similar to our initial method for measuring purity, whereas our current approach determines purity based on the entire transcriptome. This aspect, along with our focus on a DNN that distinguishes LumA from all other subtypes, contributed to 100% accuracy in identifying pure cases, as opposed to a four-class DNN with only 62% accuracy for identifying pure cases from all four major subtypes. Our emphasis on producing a continuous metric for LumA purity also presents clear implications for clinical significance for this largest subset of breast cancers. Lastly, instead of inferring tumor-rich regions by cell crowding, we used blinded manual annotation of tumor regions by an expert pathologist to avoid contamination of training and validation data with nontumor images.

In other substantively related work, Levy-Jurgenson and colleagues (35), also using TCGA images, selected eight mRNAs from the PAM50 set plus two miRNAs to train separate DNNs based on the expression of each molecular trait. Patch labels for each trait were obtained using an ensemble approach with majority voting and combined using tensor cartography to create multilayer heatmaps and to produce a metric for overall ITH. They show that tumors with higher heterogeneity for the two top-performing traits have poorer OS. Although this work supports the potential importance of spatially resolved subtype heterogeneity, the method is quite complex and relies upon a somewhat arbitrary selection of molecular traits for model training. In contrast, our simpler approach relies on intrinsic subtyping, which has well-established clinical significance, and as noted above, can be directly applied to identifying LumA cancers with higher-than-expected risk.

In addition to the strengths of our approach cited above, some limitations must also be recognized. TCGA dataset, although of high quality and sufficient to yield 230 held-out cases for the main conclusions, is still somewhat limited in size, which precluded mapping the three alternate subtypes. Several factors could have helped overcome this limitation. As noted above, preannotation by a pathologist eliminated less informative tissue areas, and the model was trained on an established method for partitioning genomic data rather assigned PAM50 or a heterogeneity metric derived from PAM50 genes alone. These factors improved the statistical efficiency of the analysis by enabling strongly supervised training and the availability of patch-level information during model development. In addition, we observed empirically that our methodology benefitted from several technical elements: the use of KimiaNet, a DNN pretrained on histomorphology rather than general images, the selection of images for parameter tuning representing a broad range of admixture, and the use of a novel modification to the cross-entropy objective function for model assessment that downweighs noisy images. A replication of our findings in additional cohorts is important, as all cases used were selected from the same TCGA database. However, generalizability was enhanced to a degree as cases were derived from multiple clinical centers, including eight used exclusively for testing, and favorable model results in the latter subset indicate that our results were not attributable to hospital-related factors acting as confounders. Furthermore, standardized H&E staining and scanning can be considered strengths in a proof-of-concept context, as potential sources of unwanted variance were controlled.

Reliance on transcriptomic data from bulk samples could have underestimated the degree of heterogeneity present in each entire tumor. Multiplex ISH, multiplex immunofluorescence, and emerging techniques for spatial transcriptomics show promise for revealing genomic diversity in WSIs and will become increasingly useful as cost barriers decrease (36). In addition to validation of our findings in other cohorts, many opportunities exist for the extension of this work. Similar models can be developed to recognize heterogeneity within other subtypes and in specific subtype combinations. Rapid analysis of H&E-stained WSIs should be evaluated as an inexpensive means to improve prediction of response to targeted therapy, as in HER2-low cases (37). Future models could incorporate multiomic data and intensive study of the molecular and cellular characteristics of divergent tumor regions, including the tumor-associated microenvironment. Finally, weakly supervised or unsupervised machine learning approaches could direct attention to specific regions or features of a tumor that induce clonal evolution and define subtype identity (38).

In conclusion, we have trained a deep learning model to recognize correlates of genomic profiles in tissue morphology from WSIs that allow us to measure ITH by subtype in breast cancer and infer its clinical consequences. Such a model has considerable advantages for deployment because of its low cost and scalability relative to other methods for evaluating spatial heterogeneity. In the clinical setting, although IHC panels can suggest subtype ambiguity, the model offers advantages over IHC in terms of both reproducibility and validity. Indeed, a recent study described a “virtual multiplexing” DNN model capable of generating images that reliably replicate multiple IHC stains based on a single H&E image (39). In research, approaches such as ours can be used as a rapid, inexpensive screen on routine H&E images to identify regions within a tumor that require in-depth study to reveal phenotypes driving prognosis or treatment response. The framework developed in this study has the potential to accelerate the development of computer-assisted precision medicine solutions that ultimately lead to more personalized and effective treatment options for patients.

## Supplementary Material

Supplementary Figure S1 Supplementary Figure S1. Scatterplot of LumA proportion by transcriptomic analysis versus percentage of tumor image patches classified as LumA by the DNN model - including held-out cases with nonLumA PAM50 assignment (n = 256). Best-fitting regression line shown, with 95% confidence band. Horizontal lines depict quartile thresholds for number of cases.

Supplementary Figure S2 Supplementary Figure S2. Paired scatterplots comparing 17 Luminal A cases from the test set obtained from hospitals that did not contribute cases for model training versus the 213 Luminal A cases remaining in the test set. Correlations between iLumA% from the image model and A. pLumA representing transcriptome purity, B. Oncotype DX score, C. Mammaprint score, and D. Expression of GRB7, which plays an important role in HER2 aggressiveness.

Supplementary Table S1 4-part table showing hospital sources for cases in the training, validation, initial testing, and final testing subsets.

Supplementary Table S2 Supplementary Table S2. Clinical and molecular features of PAM50 Luminal A breast cancers in the independent test set (n = 230) according to degree of adherence of transcriptomic profile to the LumA subtype by semi-supervised noon-negative matrix factorization (ssNMF).
