# Supplementary material for: Deep Learning Predicts Subtype Heterogeneity and Outcomes in Luminal A Breast Cancer Using Routinely Stained Whole-Slide Images
Source: Cancer Res Commun. 2025 Jan 27;5(1):157–66. doi: 10.1158/2767-9764.CRC-24-0397 (PMC11770635; doi:10.1158/2767-9764.CRC-24-0397)
Supplement: Supplementary Figure S1 — Scatterplot of LumA proportion by transcriptomic analysis versus percentage of tumor image patches classified as LumA by the DNN model - including held-out cases with nonLumA PAM50 assignment (n = 256). Best-fitting regression line shown, with 95% confidence band. Horizontal lines depict quartile thresholds for number of cases. [file crc-24-0397_supplementary_figure_s1_suppsf1.pdf]

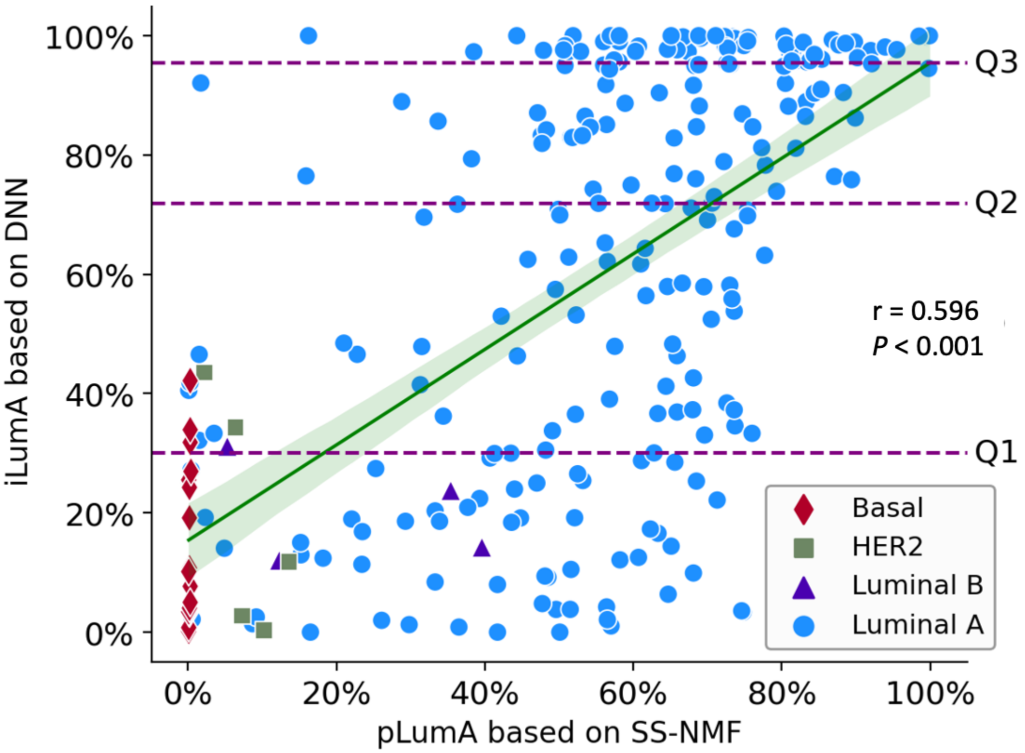

Supplementary Figure S1. Scatterplot of LumA proportion by transcriptomic analysis versus percentage of tumor image patches classified as LumA by the DNN model - including held-out cases with nonLumA PAM50 assignment (n = 256). Best-fitting regression line shown, with 95% confidence band. Horizontal lines depict quartile thresholds for number of cases.
