# Supplementary material for: Deep Learning Predicts Subtype Heterogeneity and Outcomes in Luminal A Breast Cancer Using Routinely Stained Whole-Slide Images
Source: Cancer Res Commun. 2025 Jan 27;5(1):157–66. doi: 10.1158/2767-9764.CRC-24-0397 (PMC11770635; doi:10.1158/2767-9764.CRC-24-0397)
Supplement: Supplementary Figure S2 — Paired scatterplots comparing 17 Luminal A cases from the test set obtained from hospitals that did not contribute cases for model training versus the 213 Luminal A cases remaining in the test set. Correlations between iLumA% from the image model and A. pLumA representing transcriptome purity, B. Oncotype DX score, C. Mammaprint score, and D. Expression of GRB7, which plays an important role in HER2 aggressiveness. [file crc-24-0397_supplementary_figure_s2_suppsf2.pdf]

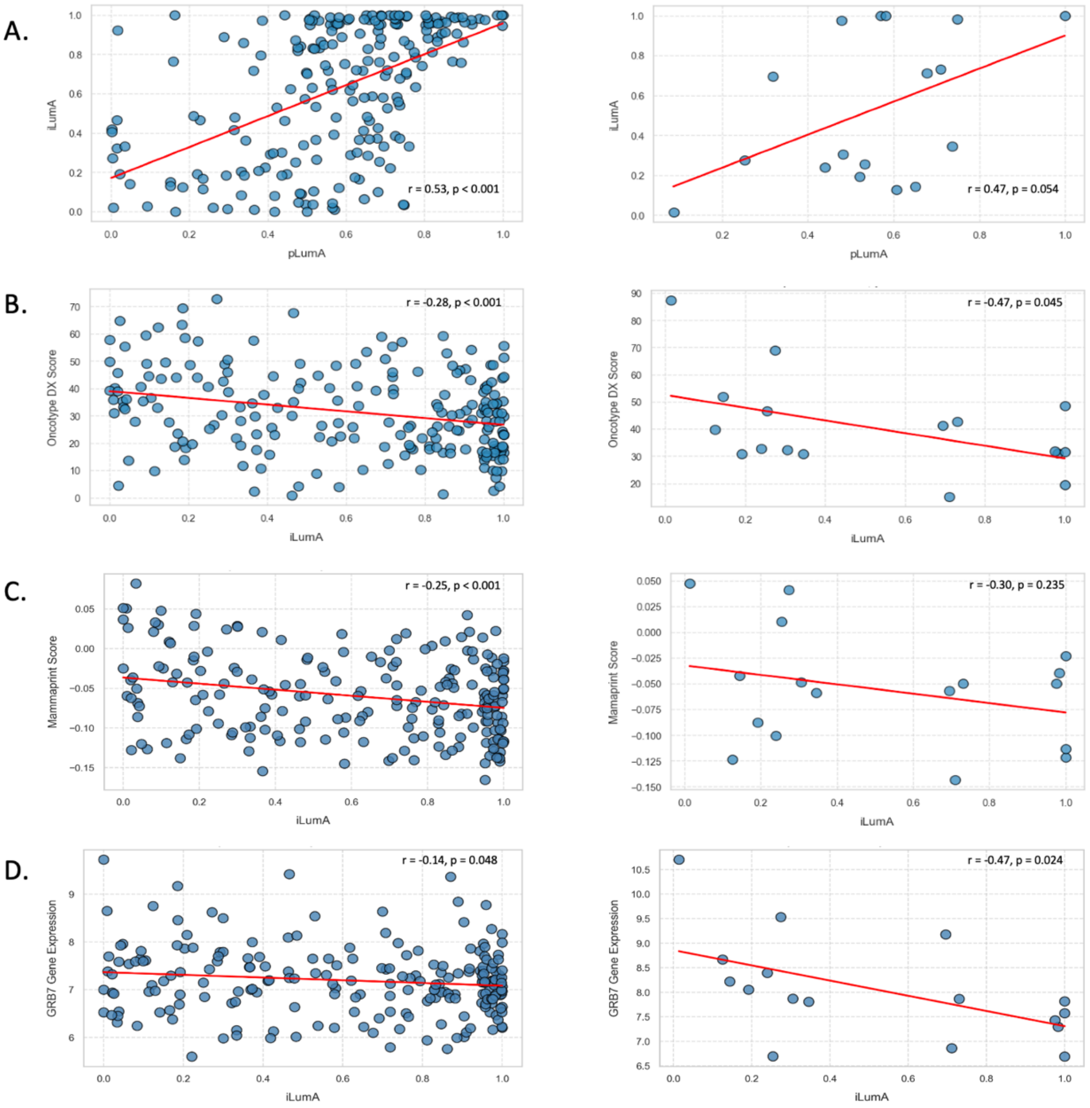

Supplementary Figure S2. Paired scatterplots comparing 17 Luminal A cases from the test set obtained from hospitals that did not contribute cases for model training versus the 213 Luminal A cases remaining in the test set. Correlations between iLumA% from the image model and A. pLumA representing transcriptome purity, B. Oncotype DX score, C. Mammaprint score, and D. Expression of *GRB7*, which plays an important role in HER2 aggressiveness.
