# Supplementary material for: Deep Learning Predicts Subtype Heterogeneity and Outcomes in Luminal A Breast Cancer Using Routinely Stained Whole-Slide Images
Source: Cancer Res Commun. 2025 Jan 27;5(1):157–66. doi: 10.1158/2767-9764.CRC-24-0397 (PMC11770635; doi:10.1158/2767-9764.CRC-24-0397)
Supplement: Supplementary Table S1 — 4-part table showing hospital sources for cases in the training, validation, initial testing, and final testing subsets. [file crc-24-0397_supplementary_table_s1_suppst1.pdf]

Supplementary Table S1A. List of center names with their codes and the number of TCGA cases used for <u>training</u> the DNN model; centers not used in testing are denoted with an asterisk.			
**Sl No:**	**Center Code**	**Hospital/Institution Name**	**Count**
1	A2	Walter Reed	20
2	A7	Christiana Healthcare	1
3	AC	International Genomics Consortium	6
4	AN	Cureline	3
5	AR	Mayo	7
6	B6	Duke	2
7	BH	University of Pittsburgh	10
8	C8	ILSBio	5
9	D8	Greater Poland Cancer Center	10
10	E2	Roswell Park	10
11	E9	Asterand	5
12	EW	University of Miami	3
13	GM	MD Anderson	2
14	LL	Candler	3
15	OL	University of Chicago	4
16	PL*	Institute of Human Virology Nigeria*	1
17	W8*	Greenville Health System*	1
18	XX*	Spectrum Health*	1
			**Total: 94**

Supplementary Table S1B. List of center names and codes, and the number of TCGA cases used for <u>validation</u> (hyperparameter tuning) of the DNN model			
**Sl No:**	**Center Code**	**Hospital/Institution Name**	**Count**
1	A2	Walter Reed	1
2	A7	Christiana Healthcare	2
3	AC	International Genomics Consortium	2
4	AN	Cureline	1
5	AR	Mayo	1
6	BH	University of Pittsburgh	6
7	C8	ILSBio	3
8	D8	Greater Poland Cancer Center	3
9	E2	Roswell Park	1
10	E9	Asterand	1
11	EW	University of Miami	1
12	OL	University of Chicago	1
			**Total: 23**

Supplementary Table S1C. List of center names and codes, along with the number of TCGA cases used for initial testing of the DNN model (Pure Test). Centers not included in training are denoted with an asterisk.

Sl No:	Center Code	Hospital/Institution Name	Count
1	A1*	UCSF*	1
2	A2	Walter Reed	12
3	A7	Christiana Healthcare	2
4	AC	International Genomics Consortium	1
5	AN	Cureline	2
6	AR	Mayo	3
7	BH	University of Pittsburgh	3
8	C8	ILSBio	3
9	D8	Greater Poland Cancer Center	4
10	E2	Roswell Park	6
11	E9	Asterand	2
12	EW	University of Miami	7
13	GM	MD Anderson	1
14	OL	University of Chicago	2
15	UU*	MBPCC*	1

Total: 50

Supplementary Table S1D: List of center names and codes, along with the number of TCGA cases used for final testing of the DNN model. Centers not included in training are denoted with an asterisk.

Sl No:	Center Code	Hospital/Institution Name	Count
1	3C*	Columbia University*	1
2	A1*	UCSF*	6
3	A2	Walter Reed	20
4	A7	Christiana Healthcare	9
5	AC	International Genomics Consortium	5
6	AN	Cureline	13
7	AQ*	UNC*	2
8	AR	Mayo	14
9	B6	Duke	13
10	BH	University of Pittsburgh	57
11	C8	ILSBio	8
12	D8	Greater Poland Cancer Center	17
13	E2	Roswell Park	29
14	E9	Asterand	11
15	EW	University of Miami	8
16	GI*	ABS – IUPU*	1
17	GM	MD Anderson	7
18	LD*	Hartford Hospital*	1
19	LL	Candler	1
20	OL	University of Chicago	1
21	S3*	Albert Einstein Medical Center*	4
22	UL*	Boston Medical Center*	1
23	WT*	University of Kansas*	1

Total: 230
