# Supplementary material for: Deep Learning Predicts Subtype Heterogeneity and Outcomes in Luminal A Breast Cancer Using Routinely Stained Whole-Slide Images
Source: Cancer Res Commun. 2025 Jan 27;5(1):157–66. doi: 10.1158/2767-9764.CRC-24-0397 (PMC11770635; doi:10.1158/2767-9764.CRC-24-0397)
Supplement: Supplementary Table S2 — Clinical and molecular features of PAM50 Luminal A breast cancers in the independent test set (n = 230) according to degree of adherence of transcriptomic profile to the LumA subtype by semi-supervised noon-negative matrix factorization (ssNMF). [file crc-24-0397_supplementary_table_s2_suppst2.pdf]

Supplementary Table S2. Clinical and molecular features of PAM50 Luminal A breast cancers in the independent test set (n = 230) according to degree of adherence of transcriptomic profile to the LumA subtype by semi-supervised noon-negative matrix factorization (ssNMF).

	**Q1 (58)**	**Q2 (57)**	**Q3 (57)**	**Q4 (58)**	**P Value (Q1 vs Q4)**	**P value (Q1-Q4)**
Age (Mean)	60.71	57.92	54.82	54.67	0.010	0.048
ER+ (%)	98.11	95.56	100	92.15	0.10	0.489
PR+ (%)	87.50	86.50	96.29	90.74	0.80	0.428
HER2+ (%)	34.38	15.90	14.28	13.15	<0.001	0.159
ER or PR + /HER2- (%)	57.14	80.00	87.8	84.21	<0.001	0.167
Node Positive (%)	62.96	50.91	55.77	46.42	0.089	0.184
Tumor Size>20mm (%)	64.91	67.76	55.00	50.87	0.04	0.114
Grade 3 + (%)	28.00	25.43	11.76	11.11	<0.001	0.065
Stage>1 (%)	80.70	74.50	66.66	58.93	0.009	0.001
Proliferation Score (Mean)	8.61	8.52	8.20	7.95	<0.001	0.019
Recurrence Score (Mean)	35.38	34.59	29.40	26.23	0.001	0.031
MammaPrint High (%)	29.31	10.52	7.01	6.89	<0.001	0.147
Mutational Load (Median)	26.0	23.0	20.0	22.0	0.14	0.225
MATH Score (Mean)	0.36	0.35	0.37	0.38	0.46	0.200
TP53 (%)	10.91	6.00	14.54	15.78	0.21	0.319
PIK3CA (%)	30.90	30.3	43.64	42.10	0.04	0.148
CBFB (%)	1.88	4.00	3.63	8.77	0.04	0.111
Oncotype DX (mean)	40.23	33.91	28.58	24.95	0.002	0.007
ER and PR+/HER2-(%)	59.18	69.56	80.00	76.59	0.014	0.120
ER Group Score (mean)	8.99	9.14	9.43	9.20	0.15	0.350
